# Compression de l'artère iliaque externe par armature de kerboull: à propos d'un cas

**DOI:** 10.11604/pamj.2015.22.251.7566

**Published:** 2015-11-18

**Authors:** Nabil Elkoumiti, Hicham El Hyaoui, Abdessalam Achkoun, Abdeljabar Messoudi, Mohamed Rahmi, Abdelhak Garch

**Affiliations:** 1 Service de Traumatologie Orthopédie, Pavillon 32, Centre Hospitalier Universitaire Ibn Rochd 1, rue des Hôpitaux, Quartier des Hôpitaux 20360, Casablanca, Maroc

**Keywords:** Prothèse de hanche, descellement, protrusion, complication vasculaire, Hip prosthesis, loosening, protrusion, vascular complication

## Abstract

Les complications vasculaires après arthroplastie totale de hanche sont exceptionnelles. Elles doivent être constamment redoutées car elles engagent à la fois le pronostic vital et le pronostic fonctionnel du membre opéré. Nous rapportons le cas d'une compression de l'artère iliaque externe diagnostiquée par angioTDM à 2 ans d'une reprise de prothése totale de la hanche, devant une symptomatologie atypique. Le mécanisme en cause était une compression de l'artère par une armature de Kerboul enfoncée en endo-pelvien. Une revue de la littérature des complications vasculaires survenant après une arthroplastie totale de hanche a permis de mettre en évidence la multiplicité des mécanismes et des présentations cliniques que peut avoir ce type de complications. La plupart de ces complications peuvent être au mieux prévenues ou traitées plus efficacement moyennant un bilan préopératoire et une surveillance postopératoire attentive.

## Introduction

Les complications vasculaires survenant après une arthroplastie totale de hanche sont exceptionnelles, chaque équipe de chirurgie orthopédique ne rapportant que quelques cas isolés sur de longues périodes. Elles restent graves car elles engagent le pronostic fonctionnel du membre opéré, voire le pronostic vital du patient. Nous rapportons un cas de compression de l'artère iliaque externe par une armature de Kerboul enfoncée en endo-pelvien.

## Patient et observation

**Figure 1:**
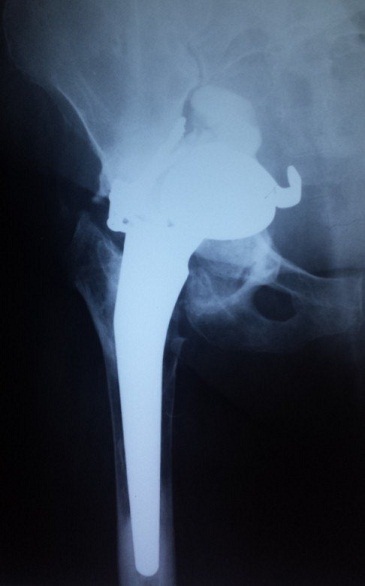
Descellement prothétique bipolaire, la pénétration intrapelvienne est sévère et concerne l'armature, la cupule et la tête prothétique

**Figure 2:**
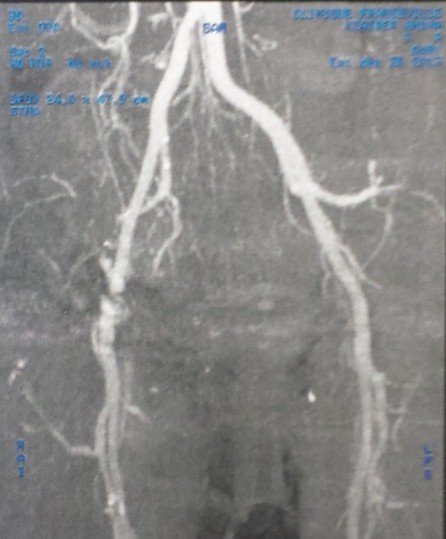
Angio-TDM montrant l'arrêt du produit de contraste au niveau de l'artère iliaque externe

**Figure 3:**
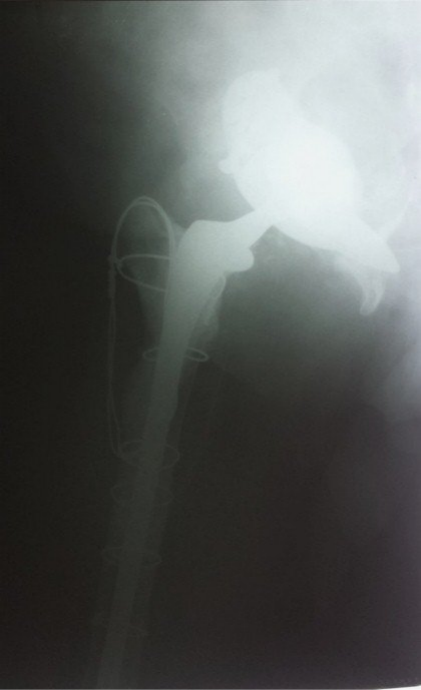
Radiographie postopératoire, prothése totale de hanche avec tige fémoeale longue et armature de Burch-Schneider

**Figure 4:**
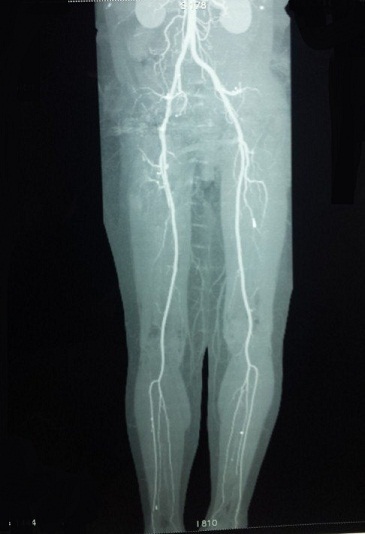
Angio-TDMde contrôle montrant la levée de la compression vasculaire

Il s'agit d'une femme âgée de 47 ans, hospitalisée dans notre établissement en Mai 2014 pour des douleurs sévères de la hanche droite, claudications intermittentes intenses avec oedème de membre inférieur droit. À l'examen clinique, il existait un enraidissement sévère de la hanche droite; le membre inférieur droit était raccourci de 3 centimètres, fixé à 35° de rotation externe et à 20° de flessum, les téguments du membre inférieur droit étaient d'apparence normale, les pouls périphériques étaient perçus mais asymétriques par rapport au côté controlatéral. Deux interventions préalables avaient été effectuées dans une autre institution par voie d'abord postéro-externe de Moore: une première arthroplastie droite scellée avait été implantée en 2010 et reprise en 2012 en raison d'un descellement ayant motivé le changement des deux composants. Les suites de ce changement avaient été marquées par une impotence fonctionnelle progressive d’évolution sévère puisqu'elle a conduit la patiente à un alitement presque complet depuis 6 mois. Les radiographies montraient un descellement bipolaire avec une protrusion intra-pelvienne sévère impliquant la croix de Kerboul, la cupule et la tête prothétique (Figure 1). Un examen angio-tomodensitométrique (angio-TDM) était pratiqué objectivant une compression de l'artère iliaque externe par la croix de Kerboul (Figure 2). La dépose de la prothèse était pratiquée en collaboration avec un chirurgien vasculaire. Il n'existait pas de faux anevrysme de l'artère iliaque externe. La cupule était progressivement libérée et déposée ainsi que le ciment et l'armature acétabulaire. L'ablation de la tige fémorale ainsi que celle du fourreau de ciment étaient faites par fémorotomie. Un deuxième temps opératoire (au cours de la même anesthésie) a consisté en une arthroplastie totale de la hanche moyennant une prothèse double mobilité avec longue tige fémorale. Le defect osseux au niveau cotyle était comblé par une armature de Burch-Schneider et une greffe osseuse prélevée de la crête iliaque (Figure 3). L'angio-TDM de contrôle montrait la levée de la compression vasculaire (Figure 4). A dix mois de l'intervention, la patiente avait retrouvé une fonction normale.

## Discussion

Les complications vasculaires lors d'une chirurgie prothétique de la hanche sont très rares. Les différentes séries de la littérature rapportent une fréquence moyenne de 0,2 à 0,3% [1]. Ces complications peuvent être immédiates: hémorragie (avec parfois hématomes compressifs) et/ou ischémie par plaie artérielle, dissection artérielle traumatique ou mobilisation de plaque d'athérome, ou retardées comme dans le cas du pseudo-anévrisme et de la fistule artérioveineuse. Ces lésions vasculaires intéressent par ordre de fréquence décroissant [2]: l'artère iliaque externe, l'artère fémorale commune, la veine iliaque externe, l'artère iliaque interne et les pédicules glutéaux. Les protrusions intrapelviennes représentent une complication rare mais sévère pouvant survenir au cours du descellement acétabulaire des prothèses totales de hanche. Elles surviennent plus fréquemment en cas d'infection. Cependant, des protrusions ont aussi été décrites après des descellements aseptiques [3], notamment en cas de défaut de technique opératoire. La protrusion est l'une des principales causes de lésions vasculaires tardives. Elle est le plus souvent progressive ce qui permet la cicatrisation et la formation d'une épaisse capsule autour des composants. Cette capsule assure une « relative » protection des structures nobles de proximité, mais des ulcérations vasculaires [4] ou d'organes pelviens [5] ont été observées de même que des symptômes en rapport avec l'inclusion des organes nobles dans cette néocapsule [6]. Ainsi le tableau clinique est plus insidieux. Dans tous ces cas, le moindre doute doit faire réaliser une exploration avec opacification vasculaire en urgence. Le cas que nous présentons ici s'inscrit parmi ce second groupe de patients aboutissant à une hypoperfusion distale. L'expression clinique d'un tel tableau d'ischémie subaiguë est souvent atypique [1].

Pour préciser au mieux les rapports et rechercher ces anomalies éventuelles au contact des implants, le meilleur examen est actuellement l'angio-TDM avec retour veineux. Il est probable que l'angio-IRM soit plus performante mais elle est encore actuellement gênée par les artéfacts liés au matériel prothétique. En tout état de cause, cet examen est capital pour guider la stratégie thérapeutique devant une migration allant à proximité des vaisseaux, cet examen permet de donner l’état du muscle ilio-psoas qui conditionne la voie d'abord pour le contrôle vasculaire et l'extraction du matériel [7]. D'autres examens sont effectués à la demande en fonction du bilan précédent et de l'existence de signes cliniques évocateurs.Ainsien cas de doute sur une lésion urinaire ou la forte proximité entre le matériel intrapelvien et voies urinaires, une uro-TDM permet de confirmer la lésion et son retentissement; une opacification digestive si l'angio-TDM objective la présence du matériel intra-pelvien menaçant les structures digestives. Une PTH avec protrusion sévère ou l'identification d'un contact étroit avec les structures nobles sur le bilan préopératoire justifient des voies d'abord électives qui permettent le contrôle et la protection des structures vasculo-nerveuses. Quatre types d'abord sont possibles: la voie sous-péritonéale [8], la voie d'abord « triradiée » de Mears [9], la voie d'abord transabdominale (laparotomie)et les voies combinées simultanées. La voie de Mears apparaît surtout adaptée aux gestes de reconstruction osseuse, mais elle ne permet pas un contrôle correct des organes nobles. Tazawa et al. [10] ont délimité les indications respectives des voies sous-péritonéale et transabdominale en fonction de l’état du muscle psoas-iliaque: si le muscle est intact, il assure une protection suffisante et la voie d'abord sous-péritonéale peut être recommandée; si le muscle ilio-psoas est détruit et si les composants sont au contact du péritoine, la voie d'abord transabdominale est préférée. L'usage systématique de la voie transabdominale peut être discutée en raison de la lourdeur de ses suites, car il est possible de faire un contrôle vasculaire correct peut être réalisé par voie sous péritonéale, mais en cas de menace directe pour les structures nobles et ou d'enfoncement intrapéritonéale, la laparotomie semble plus adaptée [7]. Chez notre patiente devant un muscle iliopsoas intact, assurant une protection suffisante, la voie d'abord sous-péritonéale a été recommandée.

## Conclusion

La prise en charge d'un descellement de PTH avec protrusion sévère et complication vasculairesuppose une évaluation précise des rapports avec les structures pelviennes. L'analyse de chaque cas doit permettre de déterminer, d'une part, l’état de la circulation artérioveineuse et d'autre part, la stratégie chirurgicale adéquate (nécessité ou non d'un abord spécifique pour contrôler les vaisseaux iliaques avant le geste orthopédique). Enfin l’évolution spontanée défavorable de cette arthroplastie descellée rappelle la nécessité d'une surveillance des arthroplasties avant la survenue de complications.
